# Visual Servoing for an Autonomous Hexarotor Using a Neural Network Based PID Controller

**DOI:** 10.3390/s17081865

**Published:** 2017-08-12

**Authors:** Carlos Lopez-Franco, Javier Gomez-Avila, Alma Y. Alanis, Nancy Arana-Daniel, Carlos Villaseñor

**Affiliations:** 1 Centro Universitario de Ciencias Exactas e Ingenierías, Universidad de Guadalajara, Blvd. Marcelino García Barragán 1421, Guadalajara C.P. 44430, Jalisco, Mexico; javier.ega@hotmail.com (J.G.-A.); alma.alanis@cucei.udg.mx (A.Y.A.); nancy.arana@cucei.udg.mx (N.A.-D.); cavp@outlook.com (C.V.); 2 Avenida Revolución 1500 Modulo “R”, Colonia Universitaria, Guadalajara C.P. 44430, Jalisco, Mexico

**Keywords:** unmanned aerial vehicle, hexarotor, visual servoing

## Abstract

In recent years, unmanned aerial vehicles (UAVs) have gained significant attention. However, we face two major drawbacks when working with UAVs: high nonlinearities and unknown position in 3D space since it is not provided with on-board sensors that can measure its position with respect to a global coordinate system. In this paper, we present a real-time implementation of a servo control, integrating vision sensors, with a neural proportional integral derivative (PID), in order to develop an hexarotor image based visual servo control (IBVS) that knows the position of the robot by using a velocity vector as a reference to control the hexarotor position. This integration requires a tight coordination between control algorithms, models of the system to be controlled, sensors, hardware and software platforms and well-defined interfaces, to allow the real-time implementation, as well as the design of different processing stages with their respective communication architecture. All of these issues and others provoke the idea that real-time implementations can be considered as a difficult task. For the purpose of showing the effectiveness of the sensor integration and control algorithm to address these issues on a high nonlinear system with noisy sensors as cameras, experiments were performed on the Asctec Firefly on-board computer, including both simulation and experimenta results.

## 1. Introduction

The use of Unmanned Aerial Vehicles (UAVs) has been increased over the last few decades. UAVs have shown satisfactory flight and navigation capabilities, which are very important in applications like surveillance, mapping, search and rescue, etc. The ability to move freely in a 3D space represents a great advantage over ground vehicles, especially when the robot is supposed to travel long distances or move in dangerous environments, like in search and rescue tasks. Commonly, UAVs have four rotors; however, having more than four gives them a higher lifting capacity. The hexarotor has some advantages over the highly popular quadrotor, such as their increased load capacity, higher speed and safety, because the two extra rotors allow the UAV landing even if it loses one of the motors. However, the hexarotor is a highly nonlinear and under actuated system because it has fewer control inputs than degrees of freedom and its Lagrangian dynamics contain feedforward nonlinearities; in other words, there are some acceleration directions that can only be produced by a combination of the actuators.

In contrast with ground vehicles, it is not possible to use sensors like encoders to estimate its position. A good alternative is to use visual information as a reference, due to the high amount of information that a camera can provide in contrast with their low power consumption and low weight. Since it is not possible to know the position of a hexarotor with common on-board sensors such as Inertial Measurement Units (IMU), some works use off board sensor systems [1,2,3,4,5]; however, this kind of control limits the application to indoor navigation and adds noise and delays because of the communication between the robot and the ground station.

For such reason, visual control of UAVs has been widely performed. Although stereo vision is extensively used in mapping applications [6,7], when used in UAV navigation like in [8,9], it requires 3D reconstruction or optical flow, which are computationally expensive algorithms. In this approach, monocular vision is used, and the feature error position in the image coordinate plane is related with the robot velocity vector that reduces this error [10,11,12,13,14,15]. Consequently, we can set the position of the robot based on the camera information to control its navigation not only in indoor environments [16,17]. Classical Image Based Visual Servo (IBVS) control stabilizes attitude and position separately [18], which is not possible for underactuated systems. In [18], an Image Based approach is used for an underactuated system but approximating the depth distance to the features.

In [19], a PID controller is implemented on an hexarotor and comparisons between quaternions and Euler angles are made. In [20], the authors propose a visual servoing algorithm combined with a proportional derivative (PD) controller. However, PID approaches are not effective on highly nonlinear systems with model uncertainties such as the hexarotor [21,22]. According to this, another approach is required. In this paper, we propose the use of a Neural Network based PID. The advantages of using neural networks to control nonlinear systems are that the controller will have the adaptability and learning capabilities of the neural network [23], making the system able to adapt to actuator faults such as loss of effectiveness, which is described in [24] and solves disadvantages of the traditional PID [25] such as uncertainties of the system, communication time-delay, parametric uncertainties, external disturbances, actuator saturations and unmodeled system dynamics, among others. If to all of these issues we add the complexity to integrate servo control algorithms with vision sensors and a neural PID in a real-time implementation, it is required to have a well-designed coordination between all of the elements of this implementation, requiring different processing stages with their respective communication architecture (software and hardware).

The rest of the paper is structured as follows: Section 2 describes the robot and its dynamics. In Section 3, the visual servo control approach is introduced. In Section 5, the design of the PID controller and weights adjustment are shown. Section 4 presents the relationship between the error signals from the visual algorithm and the control signals of the hexarotor. Section 6 and Section 7 present the simulation and experimental results of the proposed approach and its comparison with the conventional PID controller. Finally, the conclusions are given in Section 8.

## 2. Hexarotor Dynamic Modeling

The hexarotor consists of six arms connected symmetrically to the central hub. At the end of each arm, a propeller driven by a brushless Direct Current (DC) motor is attached. Each propeller produces an upward thrust and, since they are located outside the center of gravity, differential thrust is used to rotate the hexarotor. In addition, the rotation of the propellers also produces a torque in the opposite direction of the rotation of the motors; therefore, there must be two groups of rotors spinning in the opposite direction for the purpose of making this reaction torque equal to zero.

**Figure 1:**
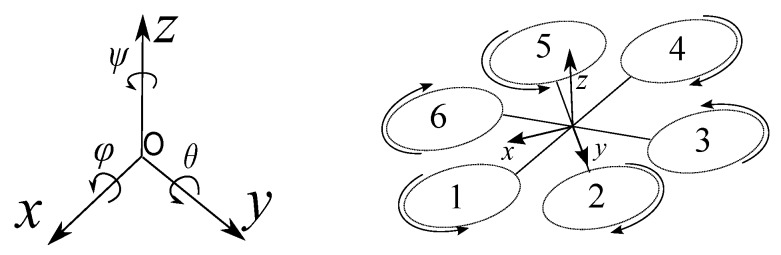
Structure of hexarotor and coordinate frames.

The pose of an hexarotor is given by its position $\zeta ={\left[x,y,z\right]}^{T}$ and its orientation $\eta ={\left[\phi ,\theta ,\psi \right]}^{T}$ in the three Euler angles roll, pitch and yaw, respectively. For the sake of simplicity, sin(·) and cos(·) will be abbreviated s· and c·. The transformation from world frame *O* to body frame (Figure 1) is given by

(1) $$\left[\begin{array}{c} x_{B}\\ y_{B}\\ z_{B} \end{array}\right]=\left[\begin{array}{ccc} c\theta c\psi & c\theta s\psi & -s\theta \\ s\phi s\theta c\psi -c\phi s\psi & s\phi s\theta s\psi +c\phi c\psi & s\phi c\theta \\ c\phi s\theta c\psi +s\phi s\psi & c\theta s\theta s\psi -s\phi c\psi & c\phi c\theta \end{array}\right]\left[\begin{array}{c} x_{W}\\ y_{W}\\ z_{W} \end{array}\right].$$

**Figure 2:**
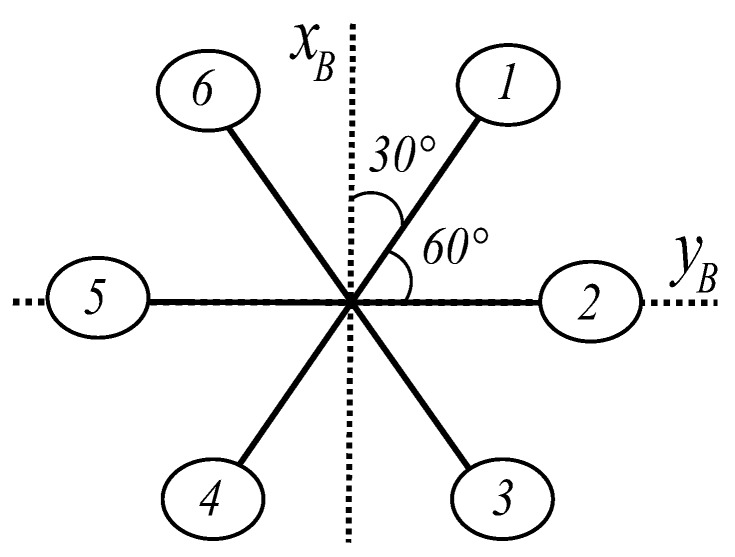
Geometry of hexarotor.

The dynamic model of the robot expressed in the body frame in Newton–Euler formalism is obtained as in [26].

(2) $$\left[\begin{array}{cc} mI_{3\times 3} & 0\\ 0 & I \end{array}\right]\left[\begin{array}{c} \dot{V}\\ \dot{\omega } \end{array}\right]+\left[\begin{array}{c} \omega \times mV\\ \omega \times I\omega \end{array}\right]=\left[\begin{array}{c} F\\ \tau \end{array}\right],$$

where *I* is the 3×3 inertia matrix; *V* the lineal speed vector and ω the body angular speed. The equations of motion for the helicopter of Figure 1 can be written as in [27]

(3) $$\begin{array}{cc} \dot{\zeta } & =v,\\ \dot{v} & =-ge_{3}+R\left(\frac{b}{m}\sum \Omega_{i}^{2}\right),\\ \dot{R} & =R\hat{\omega },\\ I\dot{\omega } & =-\omega \times I\omega -\sum J_{r}\left(\omega \times e_{3}\right)\Omega_{i}+\tau_{a}, \end{array}$$

where ζ is the position vector, R the rotation matrix from the body frame to the world frame, $\dot{R}$ represents the rotation dynamics, $\hat{\omega }$ represents the skew symmetric matrix, Ω is the rotor speed, I the body inertia, $J_{r}$ the rotor inertia, *b* is the thrust factor and τ is the torque applied to the body frame due to the rotors. Since we are dealing with an hexarotor, this torque vector differs from the well-known quadrotor torque vector and, if we are working with a structure like the one in Figure 2, it can be written as

(4) $$\tau_{a}=\left(\begin{array}{c} bl\left(-\Omega_{2}^{2}+\Omega_{5}^{2}+\frac{1}{2}\left(-\Omega_{1}^{2}-\Omega_{3}^{2}+\Omega_{4}^{2}+\Omega_{6}^{2}\right)\right)\\ \frac{\sqrt{3}}{2}bl\left(-\Omega_{1}^{2}+\Omega_{3}^{2}+\Omega_{4}^{2}-\Omega_{6}^{2}\right)\\ d\left(-\Omega_{1}^{2}+\Omega_{2}^{2}-\Omega_{3}^{2}+\Omega_{4}^{2}-\Omega_{5}^{2}+\Omega_{6}^{2}\right) \end{array}\right),$$

where *l* is the distance from the center of gravity of the robot to the rotor and *b* is the thrust factor. The full dynamic model is

(5) $$\begin{array}{cc} \ddot{x} & =\left(\cos\phi sin\theta cos\psi +\sin\phi sin\psi \right)\frac{U_{1}}{m},\\ \ddot{y} & =\left(\cos\phi sin\theta \sin\psi -\sin\phi \cos\psi \right)\frac{U_{1}}{m},\\ \ddot{z} & =-g+\left(\cos\phi \cos\theta \right)\frac{U_{1}}{m},\\ \ddot{\phi } & =\dot{\theta }\dot{\psi }\left(\frac{I_{y}-I_{z}}{I_{x}}\right)-\frac{J_{r}}{I_{x}}\dot{\theta }\Omega +\frac{l}{I_{x}}U_{2},\\ \ddot{\theta } & =\dot{\phi }\dot{\psi }\left(\frac{I_{z}-I_{x}}{I_{y}}\right)-\frac{J_{r}}{I_{y}}\dot{\phi }\Omega +\frac{l}{I_{y}}U_{3},\\ \ddot{\psi } & =\dot{\phi }\dot{\theta }\left(\frac{I_{x}-I_{y}}{I_{z}}\right)+\frac{l}{I_{z}}U_{4}, \end{array}$$

where $U_{1}$, $U_{2}$, $U_{3}$, $U_{4}$ and Ω represent the system inputs and in the case of the hexarotor are obtained as follows:

(6) $$\begin{array}{cc} U_{1} & =b\left(\Omega_{1}^{2}+\Omega_{2}^{2}+\Omega_{3}^{2}+\Omega_{4}^{2}+\Omega_{5}^{2}+\Omega_{6}^{2}\right),\\ U_{2} & =bl\left(-\Omega_{2}^{2}+\Omega_{5}^{2}+\frac{1}{2}\left(-\Omega_{1}^{2}-\Omega_{3}^{2}+\Omega_{4}^{2}+\Omega_{6}^{2}\right)\right),\\ U_{3} & =\frac{\sqrt{3}}{2}bl\left(-\Omega_{1}^{2}+\Omega_{3}^{2}+\Omega_{4}^{2}-\Omega_{6}^{2}\right),\\ U_{4} & =d\left(-\Omega_{1}^{2}+\Omega_{2}^{2}-\Omega_{3}^{2}+\Omega_{4}^{2}-\Omega_{5}^{2}+\Omega_{6}^{2}\right), \end{array}$$

where *d* is the drag factor.

## 3. Visual Servo Control

In this paper, we use an Image Based Visual Servo control approach and the eye-in-hand case. The camera is mounted on the robot and the movement of the hexarotor induces camera motion [28].

The purpose of the vision based control is to minimize the error

(7) $$e\left(t\right)=s\left(m\left(t\right),a\right)-s*,$$

where s is the vector of captured features and in the function of a vector of 2D points coordinates in the image plane, m(t) and a are the set of known parameters of the camera (e.g., camera intrinsic parameters). Vector s* contains the desired values. Since the error e(t) is defined on the image space and the robot moves in the 3D space, it is necessary to relate changes in the image features with the hexarotor displacement. The image Jacobian [29] (also known as interaction matrix) captures the relation between features and robot velocities as shown

(8) $$\dot{s}=L_{s}v_{c},$$

where $\dot{s}$ is the variation of the features position, $L_{s}$ is the interaction matrix and $v_{c}=\left(v_{c},{\dot{\omega }}_{c}\right)$ denotes the camera translational (${\dot{v}}_{c}$) and rotational (${\dot{\omega }}_{c}$) velocities. Considering $v_{c}$ as the control input, we can try to ensure an exponential decrease of the error with

(9) $$v_{c}=-\lambda {L_{s}}^{+}e,$$

where λ is a positive constant, $L_{s}\in R^{6\times k}$ is the pseudo-inverse of $L_{s}$, *k* is the number of features and e the feature error.

To calculate the interaction matrix, consider a 3D point X with coordinates (X,Y,Z) in the camera frame, the projected point in the image plane *x* with coordinates (x,y) is defined as

(10) $$\begin{array}{cc} x & =X/Z=\left(u-c_{u}\right)/f\alpha ,\\ y & =Y/Z=\left(v-c_{v}\right)/f, \end{array}$$

where (u,v) are the coordinates of the point in the image space expressed in pixel units, $\left(c_{u},c_{v}\right)$ are the coordinates of the principal point, α is the ratio of pixel dimensions and *f* the focal length. If we derive (10), we have

(11) $$\dot{x}=\frac{\dot{X}-x\dot{Z}}{Z}\;\dot{y}=\frac{\dot{Y}-y\dot{Z}}{Z}.$$

The relation between a fixed 3D point and the camera spatial velocity is stated as follows:

(12) $$\dot{X}=-v_{c}-\omega_{c}\times X.$$

Then, we can write the derivatives of the 3D coordinates as

(13) $$\begin{array}{cc} \dot{X} & =-v_{x}-\omega_{y}Z+\omega_{z}Y,\\ \dot{Y} & =-v_{y}-\omega_{z}X+\omega_{x}Z,\\ \dot{Z} & =-v_{z}-\omega_{x}Y+\omega_{y}X. \end{array}$$

Substituting (13) in (11), we can state the pixel coordinates variation as follows:

(14) $$\begin{array}{cc} \dot{x} & =-\frac{v_{x}}{Z}+\frac{xv_{z}}{Z}+xy\omega_{x}-\left(1+x^{2}\right)\omega_{y}+y\omega_{z},\\ \dot{y} & =-\frac{v_{y}}{Z}+\frac{yv_{z}}{Z}+\left(1+y^{2}\right)\omega_{x}-xy\omega_{y}-x\omega_{z}, \end{array}$$

which can be written

(15) $$\dot{x}=L_{x}v_{c}$$

with

(16) $$L_{x}=\left[\begin{array}{cccccc} -\frac{1}{Z} & 0 & \frac{x}{Z} & xy & -\left(1+x^{2}\right) & y\\ 0 & -\frac{1}{Z} & \frac{y}{Z} & 1+y^{2} & -xy & -x \end{array}\right],$$

where *Z* is the actual distance from the vision sensor to the feature, for this reason, most of the IBVS algorithms need to approximate this depth. In our case, we use an RGB-D sensor and this distance is known. To control the six degrees of freedom (DoF), at least three points are necessary [28]. In that particular case, we would have three interaction matrices $L_{x_{1}}$, $L_{x_{2}}$, $L_{x_{3}}$, one for each feature, and the complete interaction matrix is now

(17) $$L_{x}=\left[\begin{array}{c} L_{x_{1}}\\ L_{x_{2}}\\ L_{x_{3}} \end{array}\right].$$

When using three points, there are some configurations for which $L_{x}$ is singular and four global minima [30]. More precisely, there are four poses for the camera such that $\dot{s}=0$, and these four poses are impossible to differentiate [31]. With this in mind, it is usual to consider more points [28].

On the other hand, only one pose achieves s=s* when using four points. Moreover, we can use the pseudo-inverse or the transpose of the interaction matrix indistinctly to solve for $v_{c}$ in (15) [32,33].

In this paper, four points are used. In addition, our pattern does not move and because of the nature of the hexarotor, we suppose that the pattern will never be rotated since any rotation in roll or pitch will produce a translation. In other words, the hexarotor is an underactuated system, and it means there are some acceleration directions that can be only produced by a combination of the actuators. Most of the time, this is due to a lower number of actuators than degrees of freedom of the system; however, in the hexarotor, there is no actuator that can produce by itself a translational acceleration in the *x* and *y* directions. In consequence, it is not possible to have this kind of robot static and tilted at the same time, and, consequently, rotational velocities related to roll and pitch in $v_{c}$ are 0.

## 4. Control of Hexarotor

**Figure 3:**
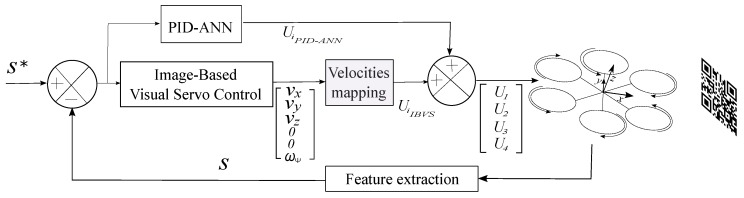
Block diagram of our Image Based Visual Servo (IBVS) control algorithm combined with Artificial Neural Network based PID (PID-ANN). The Artificial Neural Network based PID module consists of four modules (one for each Degree of Freedom). The output of the IBVS block is the velocity vector with angular velocity equal to 0 for roll and pitch since we assume the pattern will never be rotated in those angles. The output of IBVS control block consists of translational velocities $v_{x}$, $v_{y}$, $v_{z}$, which must be mapped to $U_{2}$, $U_{3}$ and $U_{1}$, respectively, in order to be added to the PID-ANN output. These $U_{i}$ control actions will be traduced to translational displacement of the robot.

The hexarotor has four control inputs $U_{i}$, $U_{1}$, which represents the translation in the *z*-axis, $U_{2}$, which represents the roll torque, $U_{3}$ the pitch, and $U_{4}$ represents the yaw torque. The visual algorithm act as a proportional controller, where λ in (9) works as a proportional gain. When combined with the Artificial Neural Network (ANN) based PID, we can adapt not only this proportional gain but also the derivative and the integral gains. Since the system is underactuated, we can use the translational velocities $\left[\dot{x},\dot{y}\right]$ computed by IBVS as input roll and pitch torques, and the error will be reduced. This is shown in Figure 3.

The velocity mapping block in Figure 3 traduces the velocities vector from IBVS to hexarotor inputs, i.e., $v_{x}=$ roll, $v_{y}=$ thrust, $v_{z}=$ pitch and $\omega_{\psi }=$ yaw. In our case, $\omega_{\phi }=0$ and $\omega_{\theta }=0$, since we assume the pattern will never be rotated because it is an underactuated system.

## 5. Neural Network Based PID

**Figure 4:**
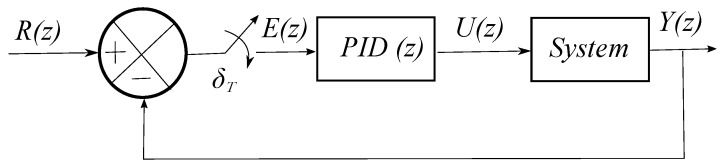
Control loop for conventional PID in discrete time.

Considering the control loop with unitary feedback as shown in Figure 4.

Conventional digital Proportional-Integral-Derivative (PID) with unitary feedback is described in [34] and the control law is given by

(18) $$U\left(z\right)=\left[K_{P}+\frac{K_{I}}{\left(1-z^{-1}\right)}+K_{D}\left(1-z^{-1}\right)\right]E\left(z\right),$$

where E(z) is the error calculated as the difference between the reference signal and the system output R(z)−Y(z). The terms $K_{P}$, $K_{I}$, and $K_{D}$ are the proportional, integral and derivative gains, respectively. These gains are related as follows:

(19) $$K_{P}=K-\frac{K_{I}}{2}\;K_{I}=\frac{KT}{T_{i}}\;K_{D}=\frac{KT_{d}}{T},$$

where *K* is the gain, *T* is the sample time, $T_{i}$ is the integration time and $T_{d}$ the derivative time. Applying the inverse *Z*-transform to (18), the PID sequence u(k) is given by

(20) $$u\left(k\right)=u\left(k-1\right)+K_{P}e\left(k\right)+K_{I}\left[e\left(k\right)-2e\left(k-1\right)+e\left(k-2\right)\right]+K_{D}\left[e\left(k\right)-e\left(k-1\right)\right].$$

Despite conventional PID being widely used to control these vehicles due to its simplicity and performance, it is not intended to control highly nonlinear systems, such as hexarotors.

**Figure 5:**
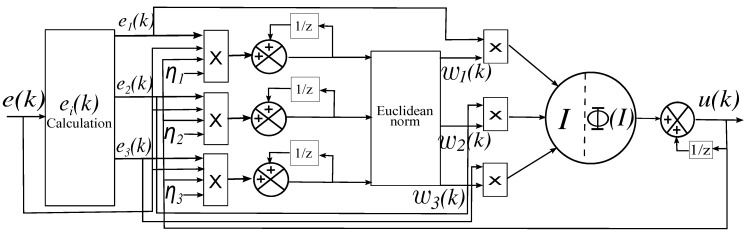
PID-ANN topology. There is one module PID-ANN for every DoF. e(k) represents the error in that DoF, $e_{i}\left(k\right)$ is a vector that represents the error, derivative of the error and the integral of the error and $\eta_{i}$ is the learning rate, which must be heuristically selected to be small enough to avoid saturation of the neuron and not necessarily the same for every gain (proportional, derivative and integral). The Euclidean norm block normalizes the neuron weights $w_{i}$ in order to avoid divergence since the neuron weights are the PID controller gains. The output of the neuron is the control input u(k) that will be traduced as thrust ($U_{1}$), roll ($U_{2}$), pitch ($U_{3}$) or yaw ($U_{4}$), depending on the state error at the input of the ANN.

In order to handle these nonlinearities, an ANN based PID controller is used. The purpose of the ANN is not only to deal with the nonlinear system, but also adjusting the PID controller gains to the end that it can also handle with uncertainties in the model. The topology of the PID-ANN used is shown in Figure 5.

From Figure 5, the $e_{i}\left(k\right)$ vector represents the proportional error, the derivative of the error and the integral of the error. They are defined as follows:

(21) $$\begin{array}{cc} e_{1}\left(k\right) & =e(k),\\ e_{2}\left(k\right) & =e(k)-2e(k-1)+e(k-2),\\ e_{3}\left(k\right) & =e(k)-e(k-1). \end{array}$$

Accordingly, the control law of the conventional PID can be rewritten as

(22) $$u\left(k\right)=u\left(k-1\right)+K_{P}e_{1}\left(k\right)+K_{I}e_{2}\left(k\right)+K_{D}e_{3}\left(k\right).$$

The Neuron input is defined as

(23) $$I=\sum_{i=1}^{3}e_{i}\left(k\right)w_{i}\left(k\right),$$

where vector $w_{i}\left(k\right)$ represents the weights of the network, which are incremented by

(24) $$\Delta w_{i}=\eta_{i}e_{i}\left(k\right)e\left(k\right)u\left(k\right)$$

with a learning factor η. The new value of $w_{i}\left(k\right)$ will be

(25) $$w_{i}^{\prime }\left(k\right)=w_{i}\left(k-1\right)+\Delta w_{i}\left(k\right).$$

The Euclidean norm will be used to limit the values of $w_{i}\left(k\right)$ as

(26) $$w_{i}\left(k\right)=\frac{w_{i}^{\prime }\left(k\right)}{∥\sum_{i=1}^{3}w_{i}^{\prime }\left(k\right)∥}.$$

The activation function of the neuron is the hyperbolic tangent; therefore, the output will be

(27) $$\Phi \left(I\right)=A\frac{1-e^{-Ib}}{1+e^{-Ib}},$$

where *A* is a gain factor to escalate the maximum value of the activation function, which is between [−1,1] and *b* is a scalar to avoid saturation of the neuron. The control law of the ANN based PID is expressed as follows:

(28) $$u\left(k\right)=u\left(k-1\right)+\Phi \left(I\right),$$

and there is one PID-ANN module for every $U_{i}$ to control in (6). $U_{i}$ has information of the rotor speed combination necessary to achieve a specific rotation, i.e., if the robot needs to move in more than one direction at the same time, there will be more than one $U_{i}$ with values different to zero.

## 6. Simulation Results

Simulations are implemented in Matlab (Matlab R2016a, The MathWorks Inc., Natick, MA, USA) using the Robotics Toolbox [35]. For the visual servo algorithm, four points are used. In the first experiment, the robot starts on the ground and has to reach a certain position given by these 2D points. With the intention of proving the algorithms, we simulate uncertainty of the system changing two parameters separately in two simulations.

**Figure 6:**
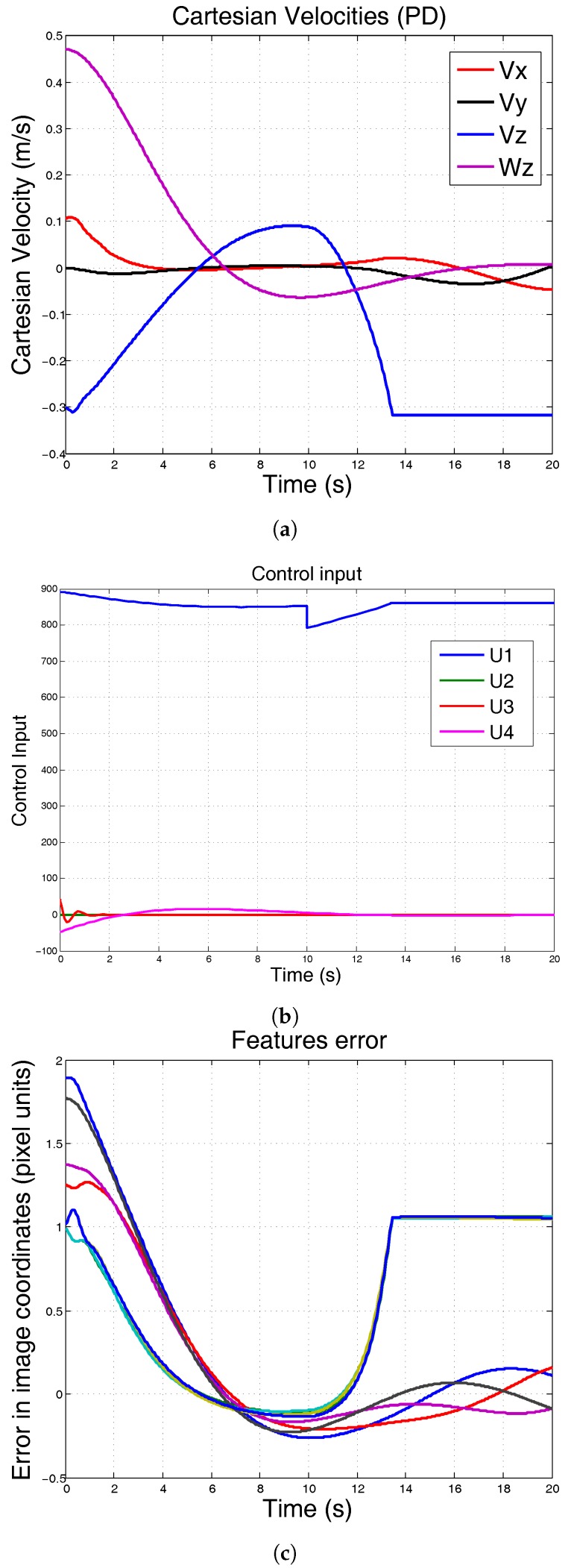
Simulation using conventional PID. At 10 s, the mass of the system is incremented 50% and λ=0.3. (**a**) Cartesian velocities; (**b**) control input; (**c**) features error.

In the first simulation, at the second 10, the mass of the robot has been increased 50%. It can be seen that conventional PID controller is unable to keep the position. The results are shown in Figure 6.

**Figure 7:**
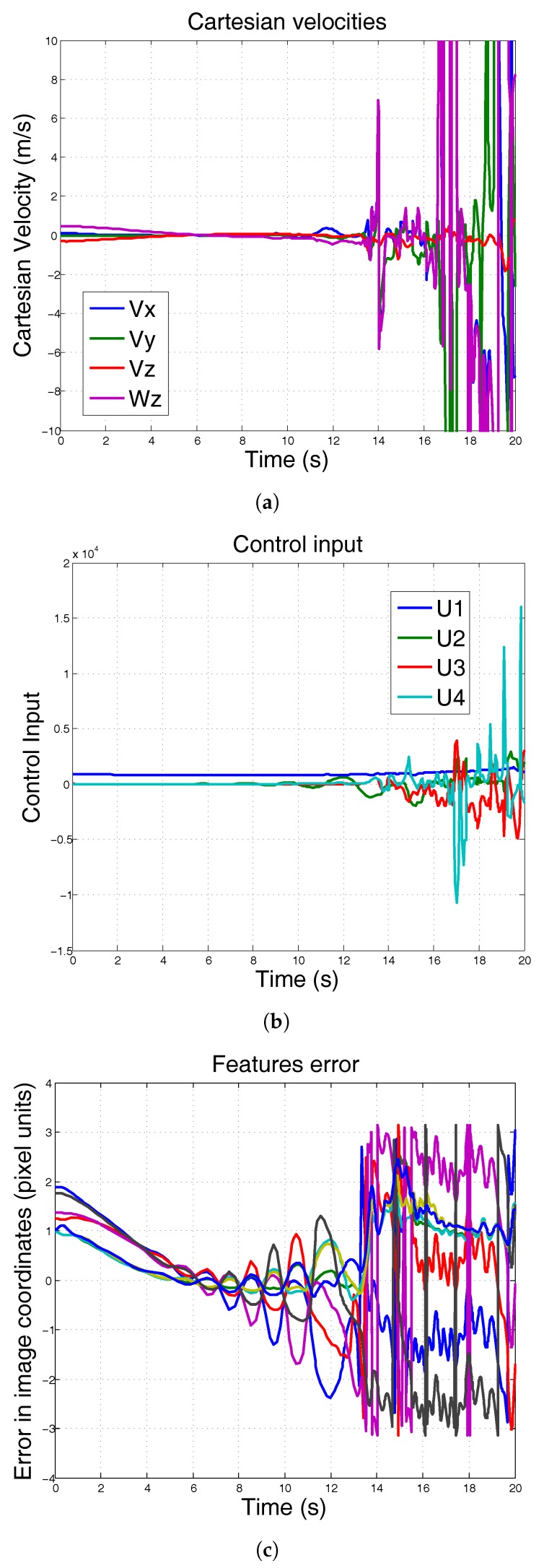
Simulation using conventional PID. Moment of inertia is increased. Mass remains constant. It can be seen that the robot is not stable when $I_{x}$ changes. (**a**) Cartesian velocities; (**b**) control input; (**c**) features error.

In the second simulation, the mass of the system remains constant, but the moment of inertia $I_{x}$ is increased from $I_{x}=0.0820$ to $I_{x}=0.550$. Figure 7 shows the results of using conventional PID when the moment of inertia is increased. The results show that the control input is excessively high to the robot (Figure 7b), making the system unable to follow the reference (Figure 7c).

**Figure 8:**
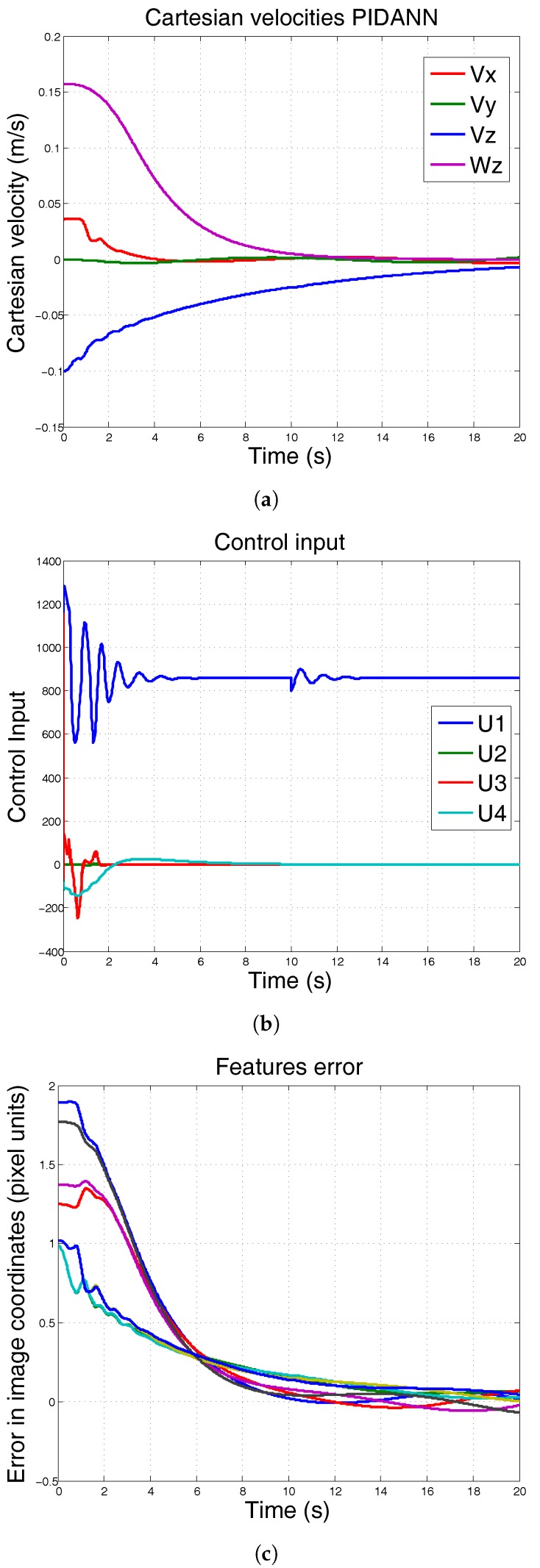
Simulation using conventional PID-ANN. At 10 s, the mass of the system is incremented 50% and λ=0.3. The system remains at desired position. (**a**) Cartesian velocities; (**b**) control input; (**c**) features error.

In the following simulations, the PID-ANN is now controlling the system under the same conditions. The mass has been incremented at second 10. It can be seen in Figure 8 that the controller can be adapted to this mass increment and keep the reference.

**Figure 9:**
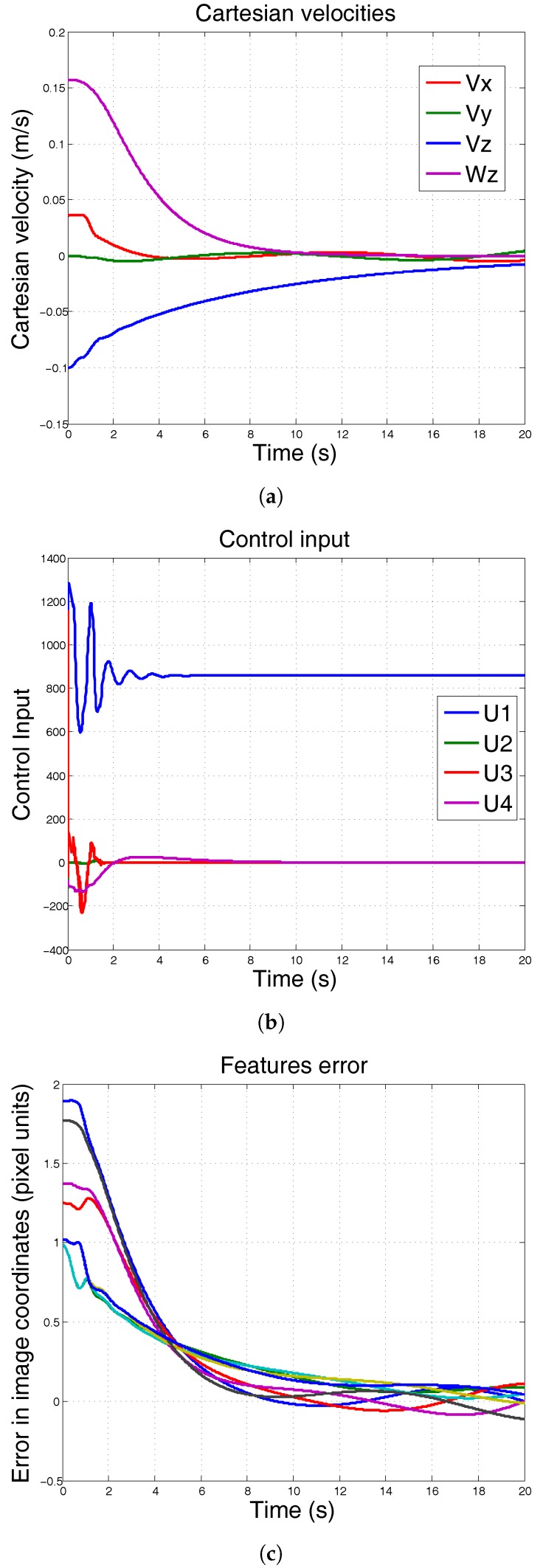
Simulation using ANN based PID. Moment of inertia is increased. Mass remains constant. It can be seen that the robot remains stable at desired position when $I_{x}$ changes. (**a**) Cartesian velocities; (**b**) control input; (**c**) features error.

Finally, we show, in Figure 9 the results of changing the moment of inertia $I_{x}$ while mass remains constant. In contrast with conventional PID, the PID-ANN is able to keep its position.

## 7. Experimental Results

**Figure 10:**
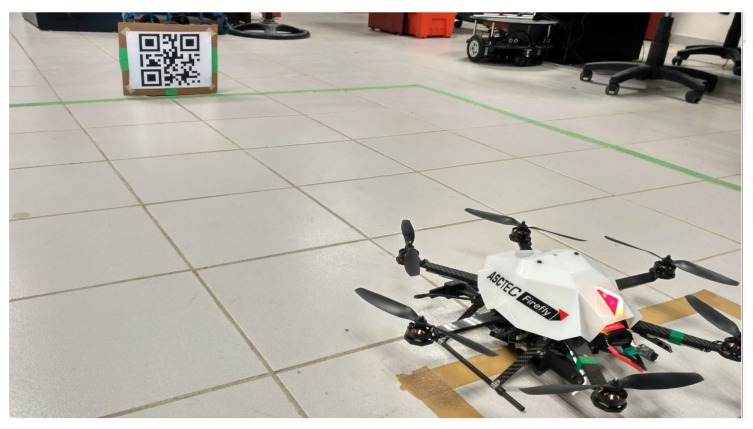
Actual experiment configuration. The corners of the Quick Response (QR) code represent the 3D features.

The hexarotor used in the experiments is the Asctec Firefly (Ascending Technologies, Krailling, Germany). The actual configuration of the experiment is shown in Figure 10. The vision sensor has been changed, we use the Intel RealSense R200 camera (Intel, Santa Clara, CA, USA) with RGB and infrared depth sensing features and an indoor range from 0.4 m to 2.8 m. This change in the vision sensor will modify robot mass and moment of inertia. This uncertainty can be absorbed by the neural network.

Vision information is highly noisy and presents a high computational cost even when working with low resolution images (in this case, 640×480). The more time the algorithm uses in image capture and processing phase, the more error will exist between what the robot sees and the actual position. Computer vision algorithms, such as optical flow approaches, requires tracking of a set of *n* features using some kind of descriptor. Other approaches use stereo vision but that requires a 3D reconstruction. We propose to use only four points to reduce this time.

It is important to note that coordination between vision sensors, neural network and model system working at different processing stages and its communication at their respective architectures is crucial to achieve real-time implementation. A QR-code is used, and we track it with a Zbar bar code reader library. This pattern is chosen because of its robustness to rotations and illumination changes. The algorithms are implemented on the onboard computer of the hexarotor.

**Figure 11:**
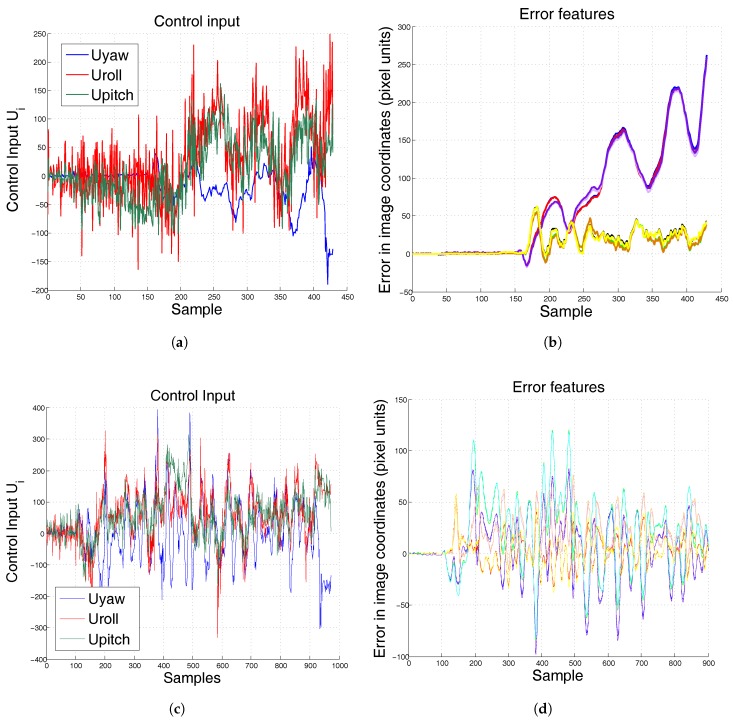
Experimental results when mass and moment of inertia changed. The pattern remains at the same position during the test. (**a**) control input conventional PID; (**b**) error conventional PID; (**c**) control input ANN-PID; (**d**) error ANN-PID.

For a first experiment, the moment of inertia and mass of the system changed and a previously tuned conventional PID controller will be compared with the proposed algorithm. Figure 11 shows results when the pattern is fixed at a certain position and the hexarotor is at hover position. As can be seen in Figure 11b, the conventional controller cannot achieve system stabilization at a fixed position when the model changes. Table 1 shows the Root Mean Square Error (RMSE) and the Average Absolute Deviation (AAD) in pixel units. The pair $\left(x_{i},y_{i}\right)$ is the location of feature (i=1,2,3,4) in image coordinates.

In Figure 11b, the solid increasing lines represent the *x* position of the four features in image coordinates (pixel units). As can be seen, if a conventional PID is not correctly tuned for this specific system, its position diverges. On the other hand, when the system is controlled by the PID-ANN, its position does not diverge (Figure 11d) even when the controller has not been previously tuned.

**Figure 12:**
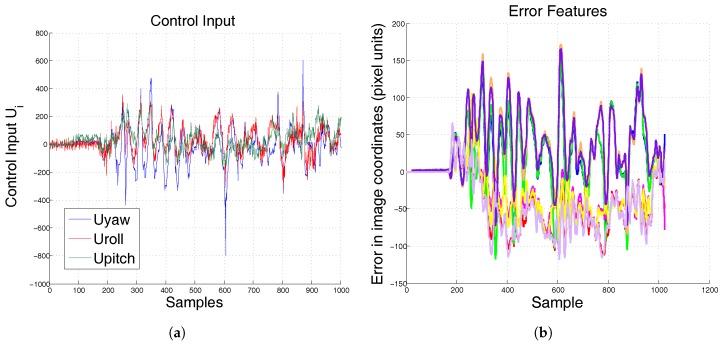
Experimental results at hover position when mass and moment of inertia changed. The pattern is moving during the test. (**a**) control input ANN-PID; (**b**) error ANN-PID.

Once ANN-PID has demonstrated its effectiveness over the PID controller, the experiment is repeated, but now the QR pattern has movement. As shown in Figure 12, the hexarotor does not lose sight of the objective.

## 8. Conclusions

In this paper, we propose a Neural Network based PID controller with visual feedback to control an hexarotor. The hexarotor is equipped with an RGB-D sensor that allows for estimating the feature error, this error has been used to compute the camera velocities. The proposed approach is able to deal with delays due to image processing, system uncertainties, noises and changes in the model since the ANN is continuously adapting the PID gains. In contrast with conventional PID controllers, where it is mandatory to tune it according to a specific system, the ANN can deal with nonlinearities and changes in the system.

## Figures and Tables

**Table 1:** Controllers’ comparison.

	**Root Mean Square Error (Pixel Units)**							
	$x_{1}$	$y_{1}$	$x_{2}$	$y_{2}$	$x_{3}$	$y_{3}$	$x_{4}$	$y_{4}$
PID	1741.9	393.1	1712.0	339.1	1677.5	329.2	1723.9	380.1
ANNPID	212.416	66.549	191.1447	57.4298	824.085	59.7654	861.8207	62.6225
	**Average Absolute Deviation (Pixel Units)**							
	$x_{1}$	$y_{1}$	$x_{2}$	$y_{2}$	$x_{3}$	$y_{3}$	$x_{4}$	$y_{4}$
PID	55.2177	9.6437	54.0636	10.5457	52.4827	10.704	53.069	9.6577
ANNPID	43.2281	7.4259	42.5686	11.3874	46.6832	11.484	47.5576	7.5503

Comparison Table between conventional Proportional Integral Derivative (PID) controller and the Artificial Neural Network based PID controller (ANNPID).

## Abbreviations

The following abbreviations are used in this manuscript:

UAV	Unmanned Aerial Vehicle
IBVS	Image Based Visual Servo
VTOL	Vertical Take-Off and Landing
PID	Proportional Integral Derivative
ANN	Artificial Neural Network
IMU	Inertial Measurement Unit
PD	Proportional Derivative
RMSE	Root Mean Square Error
AAD	Average Absolute Deviation
